# Bone Biomarkers in Mucopolysaccharidoses

**DOI:** 10.3390/ijms222312651

**Published:** 2021-11-23

**Authors:** Akari Nakamura-Utsunomiya

**Affiliations:** 1 Department of Pediatrics, Hiroshima Prefectural Hospital, 1-5-54 Ujina-Kanda, Minami-ku, Hiroshima 734-8551, Japan; a-utsunomiya@hph.pref.hiroshima.jp; Tel.: +81-82-254-1818; Fax: +81-82-253-8274; 2 Division of Neonatal Screening, Research Institute, National Center for Child Health and Development, Tokyo 157-8535, Japan; 3 Department of Pediatrics, Graduate School of Biomedical and Health Sciences, Hiroshima University, Hiroshima 734-8551, Japan

**Keywords:** bone metabolism, biomarker, mucopolysaccharidoses, chondrocyte, bone remodeling, inflammation

## Abstract

The accumulation of glycosaminoglycans (GAGs) in bone and cartilage leads to progressive damage in cartilage that, in turn, reduces bone growth by the destruction of the growth plate, incomplete ossification, and growth imbalance. The mechanisms of pathophysiology related to bone metabolism in mucopolysaccharidoses (MPS) include impaired chondrocyte function and the failure of endochondral ossification, which leads to the release of inflammatory cytokines via the activation of Toll-like receptors by GAGs. Although improvements in the daily living of patients with MPS have been achieved with enzyme replacement, treatment for the bone disorder is limited. There is an increasing need to identify biomarkers related to bone and cartilage to evaluate the progressive status and to monitor the treatment of MPS. Recently, new analysis methods, such as proteomic analysis, have identified new biomarkers in MPS. This review summarizes advances in clinical bone metabolism and bone biomarkers.

## 1. Introduction

The mucopolysaccharidoses (MPS) are a family of lysosomal storage disorders characterized by a deficiency of enzymes that degrade glycosaminoglycans (GAGs) [1]. The accumulation of GAGs in bone and cartilage leads to progressive damage in cartilage that, in turn, reduces bone growth by the destruction of the growth plate, incomplete ossification, and growth imbalance [2]. The occurrence of musculoskeletal symptoms is characteristic for all types of the disease, except for MPS IIIB. Indeed, endochondral bone growth is known to be abnormal in 6 of the 11 types of MPS disorders [3]. Abnormal development of the vertebrae and long bones is a hallmark of skeletal diseases, including several MPS [1]. Among them, dysostosis multiplex in the context of MPS is a characteristic finding and was hypothesized to be associated with abnormalities in bone remodeling given its progressive nature [4,5,6]. In addition, results with MPS animal models have suggested that bone remodeling might be impaired. Furthermore, it was hypothesized that GAG accumulation impairs bone cellular function because GAG accumulation was reported in bone cells (osteoblasts, osteoclasts, and chondrocytes) in some MPS animal models [7,8,9,10] and in a human case report [11,12]. There are also previous reports of occasional fractures and osteopenia in individuals with MPS [13,14,15]. Moreover, increased inflammatory biomarkers in MPS result in impaired bone function and poor bone tissues. Although some of the pathophysiology has been reported, precise bone biomarkers to evaluate and monitor the condition of the bone system in MPS have not yet been developed [3,16].

This review discusses recent studies of clinical bone metabolism and bone markers and summarizes recent advances in the pathophysiology and biomarkers related to the bone system in MPS.

## 2. Physiology of Normal Bone Development and Remodeling

Normal bone homeostasis is maintained by a balance between osteoblast and osteoclast activity [17]. The skeletal lineage of cells includes osteoblasts, osteocytes, and chondrocytes [17]. These are involved in the formation of bone and cartilage, whereas the osteoclasts that are responsible for bone resorption are derived from the hematopoietic lineage [17]. Osteoblasts in the craniofacial region originate from neural crest cells derived from the neural ectoderm [18]. On the other hand, the long bones of the skeleton originate from the paraxial mesoderm and lateral plate mesoderm. When bone elongation occurs, chondrocytes and osteoblasts interact with transcriptional and genetic factors in the growth plate and transient cartilaginous structure that is transformed into bone [19,20].

Chondrocytes in the growth plate consist of three distinct morphological zones (resting, proliferative, and hypertrophic), reflecting their functional properties. Proliferating chondrocytes divide and form columns parallel to the axis of growth in long bones of the limbs and vertebral columns. When chondrocytes differentiate, they subsequently undergo hypertrophic expansion into the hypertrophic zone (HZ) and produce a matrix conducive to new bone deposition [17,18,19]. Eventually, differentiated chondrocytes undergo apoptosis. This temporal and spatial sequence of events as cartilage is transformed into bone is termed endochondral ossification (EO). Chondrocyte hypertrophic differentiation is regulated by an orchestrated pattern of transcriptional factors and their signaling pathways, including SOX9, fibroblast growth factors (FGFs), bone morphogenetic proteins (BMPs), Wingless/integrated (Wnts), Indian hedgehog (IHH), and others [20,21,22]. These pathways form an interdependent signaling axis extending from the perichondrium to the growth plate, in which various secreted and soluble growth factors tightly regulate the pace of chondrocyte differentiation.

In healthy bone systems, bone is constantly being remodeled, first being resorbed (bone resorption) and then being rebuilt (bone formation) [23,24]. Bone formation is normally coupled to bone resorption so that the bone mass does not consequently change. Indeed, bone diseases occur when formation and resorption are uncoupled. In addition, bone tissue is composed of a collagen matrix on which calcium and phosphate are deposited in the form of hydroxyapatite. As chondrocytes become mature, they produce various extracellular matrix (ECM) proteins, including collagen. Collagen is deposited in a lamellar fashion and strengthened by many crosslinks. These crosslinks are pyridinolines that are resistant to degradation and are released during bone resorption in either free or peptide form [25].

Osteoblasts, the main cells responsible for bone formation, secrete extracellular matrix proteins, such as type 1 collagen, osteopontin, osteocalcin, and alkaline phosphatase [17]. Bone-specific alkaline phosphatase (BSAP) and amino-terminal pro-peptide of type I procollagen (PINP) are the most clinically useful markers of bone formation, whereas urinary N-telopeptide crosslink (NTX) and serum C-telopeptide crosslink (CTX) are widely regarded as the most clinically useful markers of bone resorption [26,27].

The serum concentrations of BSAP and osteocalcin reflect the cellular activity of osteoblasts [26,27,28,29]. The serum concentrations of the carboxy-terminal and amino-terminal pro-peptides of type I procollagen (PICP and PINP, respectively) reflect changes in the synthesis of new collagen. Fink et al. have reported that the PINP measurement appears to be more specific than PICP for the synthesis of bone collagen [30].

Urinary and serum concentrations of collagen crosslinking reflect bone resorption but not dietary intake. As a result, these are better indicators of bone resorption than urinary calcium or hydroxyproline excretion [30]. Furthermore, because deoxypyridinoline (D-PYR) and the peptide-bound alpha-1 to alpha-2 NTX and CTX are almost exclusively derived from collagen in bone, measurements of these are specific markers of bone resorption [27].

## 3. Pathophysiology Related to Bone Metabolism in MPS 

### 3.1. Accumulation of GAGs Impairs Chondrocyte Function 

**Figure 1:**
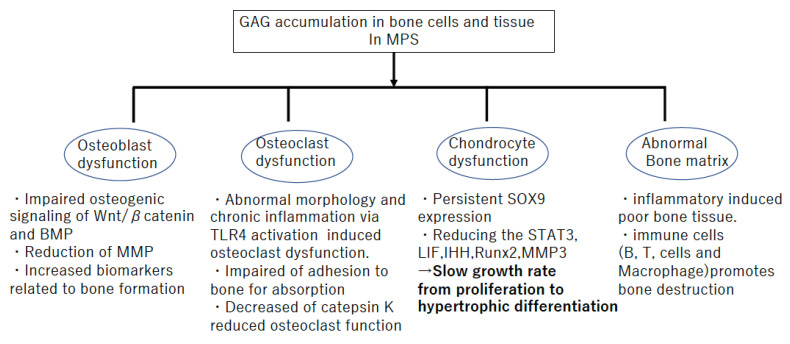
Bone pathophysiology in Mucopolysaccharidosis (MPS).

Several previous reports have demonstrated that growth plates in MPS commonly exhibit abnormalities, including enlarged and vacuole-filled chondrocytes, disorganized columnar architecture in the PZ and HZ, and reduced calcification of cartilage tissue [28,31,32] (Table 1). Similar histopathological findings were observed in animal models of MPS. Growth plate chondrocyte vacuolation and disorganized columnar structures were observed in MPS I [1,32], MPS VI [33,34,35], and MPS VII in dogs, cats, and mice [36,37,38,39,40], whereas chondrocyte vacuolation was reported in MPS IIIA and IVA mice [41]. It has been reported that the lengths of long bone and vertebrae are reduced in MPS VII, and the developmental delay in the primary and secondary centers of ossification was detected [42]. In MPS, it is assumed that the persistence of SOX9 induces delayed hypertrophic differentiation and decreased activation of signal transducer and activator of transcription 3 (STAT3) results in reduced chondrocyte proliferation [36] (Figure 1). These were suggested as molecular mechanisms underlying the poor growth plate function [43,44,45,46,47,48]. Moreover, MPS VII chondrocytes have been reported to be less able to transit from the G1 to S phase in their cell cycle; therefore, they are less able to progress to mitosis or to exit the cell cycle [49,50]. Consequently, there are fewer PZ and HZ cells in a growth plate in MPS. The typical delay of slowly growing growth plates is presented, rather than the grossly abnormal growth plate structure and function observed in some other forms of genetic skeletal dysplasia [50] (Figure 1).

### 3.2. Failures of Endochondral Ossification

**Figure 2:**
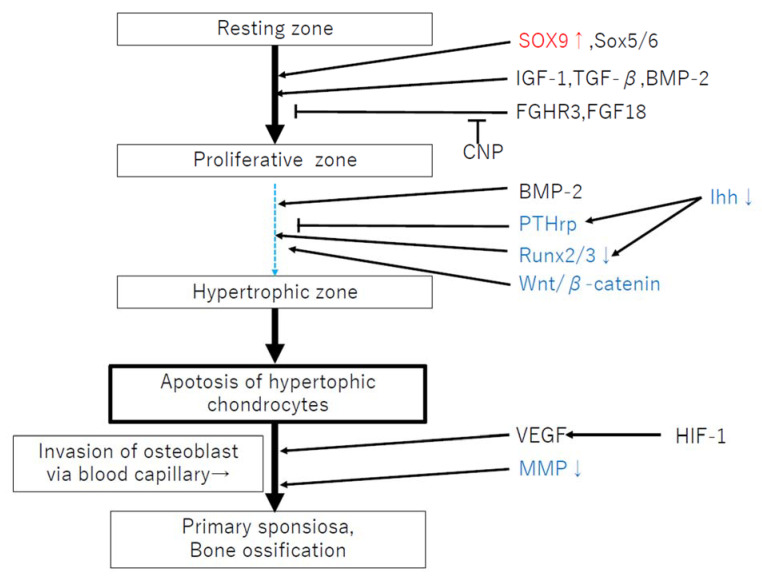
Failure of endochondral ossification related to factors in MPS. Red: increased expression, Blue: decreased expression. This figure is referred to [20].

The mechanisms involved in short stature and skeletal deformity in MPS are assumed to result from a primary failure of cartilage-to-bone conversion in the primary ossification center (POC) and secondary ossification center (SOC), growth plate dysfunction, and abnormal BMD [42]. Delayed POC and SOC formation has been observed in MPS VII mice and dogs [43]. The highly orchestrated regulation of endochondral ossification involves a complex network of systemic factors, transcription factors, and soluble growth factors in the epiphyseal cartilage and the growth plates [44,45,46,47,48]. In MPS, it has been suggested that the delayed POC and SOC formation is due to altered hypertrophic differentiation resulting from persistent SOX9 expression [42,51], reduced expression of tartrate-specific acid phosphatase and matrix metalloproteinases (MMPs), and impaired osteogenic signaling through pathways, such as Wnt/β-catenin and BMP [46,47,48,49,50,51] (Figure 2). Moreover, the accumulation of high levels of GAGs in MPS cells and tissues may, therefore, affect the activities of these secreted factors. For example, it is known that extracellular heparan sulfate has an influence in regulating the distribution and activity of secreted growth factors, including fibroblast growth factor (FGF), Wnt, and Indian hedgehog (Ihh), with a relative binding affinity for these molecules [51,52]. Bellesso et al. reported that FGF signaling and bone development were perturbed in mouse and zebrafish models of MPS II [31]. Chondrocytes in MPS VII mouse growth plates have also been reported to exhibit reduced proliferation rates, which may be attributed to the reduced expression of tyrosine phosphorylation of STAT3, leukemia inhibitory factor (LIF), and Indian hedgehog (IHH) [28,53,54]. SOX9 suppresses the expression of Runt-related transcription factor 2 (Runx2) and β-catenin signaling [51]; it has been speculated that the delays in the transition from proliferation to hypertrophic differentiation in murine MPS VII growth plates might be caused by reduced RUNX2 expression, persistent SOX9 expression, and altered PTHrP and WNT5A signaling [51].

### 3.3. Accumulation of GAGs Lead to the Release of Inflammatory Cytokines via the Activation of TLR4

GAGs have an influence on various proteins involved in physiological and pathological processes, including chemokines, cytokines, growth factors, morphogens, enzymes, and adhesion molecules [52]. It was reported that GAG storage in animal models of MPS leads to inflammation and apoptosis within the cartilage [52]. In MPS disorders, previous reports showed that the joints and bone tissues are infiltrated by proinflammatory cytokine-producing osteoclast precursors and other macrophage lineages that release proinflammatory cytokines, resulting in chronic inflammation and bone loss [52]. It is known that tumor necrosis factor (TNF-α) and other inflammatory cytokines, such as interleukin (IL-1β), are released from chondrocytes, resulting in apoptosis [53]. In addition, it is known that matrix metalloproteinases (MMPs) are released, contributing to joint and bone destruction [53]. Peck et al. indicated that lipopolysaccharide (LPS), a molecule that is structurally similar to GAGs, stimulates a signaling pathway that is critical in the pathogenesis of many chronic inflammatory diseases, including rheumatoid arthritis (RA) [54]. LPS signaling is mediated through Toll-like receptor 4 (TLR4), inducing the release of TNF-α and other proinflammatory cytokines. In MPS, it is assumed that the chronic inflammation in the joints is similar to that in RA.

Impaired STAT transcription factors and reduced autophagy are known to be altered by inflammation [35]. Hyaluronan fragments and oligosaccharides released from the degradation of the ECM were shown to signal through TLR4 via MyD88 and CD44, providing further support for the concept that GAG storage in MPS activates this pathway [55]. TLR4 activation in MPS animals resulted in the production of the proapoptotic lipid, ceramide, and the release of abundant inflammatory cytokines and proteases [35,56]. Therefore, it is assumed that the release of inflammatory cytokine via the activation of TLR4 is a key pathophysiological mechanism in bone disorders in MPS. Moreover, GAGs induce the clonal expansion of several immune cell types, including B and T cells, and macrophages; this, in turn, promotes inflammation and bone destruction [56]. This immunological and hematological background related to chronic inflammation by the accumulation of GAGs is speculated to induce damage in the growth plate and cartilage in MPS.

## 4. Biomarkers Related to Bone Metabolism in MPS 

There is an unmet need for precise biomarkers other than GAGs to evaluate the bone condition and monitor the treatment in MPS disorders. Previous research has been reported related to biomarkers in MPSI, II, IVA, VI and VII Among them, MPS IVA showed severe and distinct skeletal abnormalities because KS accumulated cartilage in addition to cornea and heart valve membrane [57]. Conventional markers, such as KS, KS sulfation levels, and chondroitin-6-sulfate levels, and the presence of collagen type II in the blood are potential biomarkers associated with bone and cartilage disease in MPS IVA [57]. Regarding bone remodeling biomarkers, osteocalcin was found to be increased in children with MPS disorders, and BSAP and urinary PYD showed an increasing trend, which suggested increased osteoblast activity. Plasma IL-1β, TNF-α, and D-PYR (which were higher in MPSIthan in controls [58] and IL-6 might also be appropriate biomarkers to monitor progression in joint contracture, short stature, and hip dysplasia over time [59] (Table 2).

Sun et al. examined the expression of selected markers and suggested chondrocyte maturation markers as a function of disease state [42]. Positive markers of chondrocyte maturation, including forkhead box protein A2 (FOXA2), were expressed at significantly lower levels in MPS VII than in controls. In addition, bone formation markers, such as alkaline phosphatase (*ALPL*), osteocalcin (*BGLAP*), collagen type 10 A1 (*COL10A1*), and osteopontin (*SPP1*), were lower in MPS VII than in controls [42] (Table 2). This phenomenon might be caused by increased and persistent expression of SOX9 [42]. The change in these biomarkers consistently suggested the failure of the chondrocyte to be hypertrophic, consequently leading to delayed bone formation in MPS.

Patel et al. recently reported that urinary-free hydroxylated (Lys-OH) and glycosylated hydroxylysines (Lys-O-Gal and Lys-O-GalGlc) in MPS patients measured by tandem liquid chromatography-tandem mass spectrometry assay were indicators of altered collagen degradation [59]. These are unique and specific post-translational modifications of collagen [63]. Lys-O-Gal is predominantly found in bone tissues, and Lys-O-GalGlc is mainly found in the skin [65]. The ratio of Lys-O-Gal to Lys-O-GalGlc has also been examined as an indicator of the source of collagen degradation; it was found to be significantly higher in all pediatric MPS I, II, and IV patients, with a small significant increase in adult MPS IV patients [59] (Table 2). However, the authors concluded that the collagen degradation products originated from a source other than bone, such as cartilage or connective tissue [59].

Sinonaro et al. reported several proinflammatory cytokines, nitric oxide, and MMPs (MMP-2, -9, and TIMP-1) as novel therapeutic targets and/or biomarkers of MPS joint and bone disease [66]. They also suggested that the cartilage of MPS patients is similar to that in those with osteo or rheumatoid arthritis [66]. Furthermore, there are reported similarities in the secretion of proinflammatory cytokines, such as TNF-α and IL-1β, nitric oxide (NO) production, apoptosis of chondrocytes, and reduced proteoglycan content in the extracellular matrix (ECM). Because MMPs are key enzymes involved in ECM degradation, and enhanced MMP activity is an important feature of arthritis, the authors speculated that these biomarkers might be used to monitor the disease severity and efficacy of treatment in MPS patients [66] (Table 2).

Jose et al. identified four potential protein biomarkers in MPS IVA, all of which may influence bone and cartilage metabolism [62]. The authors demonstrated that four proteins (fetuin-A, vitronectin, alpha-1-antitrypsin, and clusterin) might be considered biological age markers in MPS IVA. Another study reported alpha-1-antitrypsin as a candidate for a biomarker in MPS IVA [67]. Alpha-1-antitrypsin is a protease inhibitor of the proteolytic enzyme elastase produced in response to stimuli, such as inflammatory mediators [67]. Moreover, they speculated that it may be an indicator of disease progression in MPS IVA. Martell et al. also identified lipoprotein (a) and serum amyloid, in addition to alpha-1-antitrypsin, as candidates [67] (Table 2).

Fetuin-A is a glycoprotein family member structurally related to the cystatin-like protein domain and involved in osteogenesis, bone resorption, and the control of calcium salt precipitation in blood [68,69]. This protein accumulates in the bone matrix and inhibits the transforming growth factor, which is necessary for bone mineralization [69]. Fetuin-A knock-out mice showed stunted long bone growth, likely resulting from premature mineralization of growth plates [70,71]. Decreases in the plasma levels of fetuin-A may occur in response to inflammatory processes, such as those that occur in MPS IVA.

Vitronectin (VTNC), a glycoprotein in the ECM that circulates in the blood, participates in the remodeling of the extracellular matrix (ECM) and osseous integration mechanisms and regulates the proteolytic degradation of the matrix [72]. In addition, VTNC binds to the complement system, heparin, and thrombin–antithrombin III complexes, facilitating the regulation of the immune response and clot formation [72]. Crysterin (CLUS) helps to prevent cell stress-induced apoptosis and participates in the aggregation of blood plasma proteins, such as apolipoprotein C-II. It also inhibits the formation of amyloid fibrils [62] (Table 2).

Although these potential biomarkers require verification, molecules related to inflammation and degradation of the ECM might be candidate bone biomarkers. Further work is also required to understand their underlying pathological association with MPS and to develop useful biomarkers to monitor the response to treatment and clinical outcomes.

## Figures and Tables

**Table 1:** Summary of GAG accumulation and bone findings in each type of MPS.

	Accumulating GAGs	Bone Finding in Previous Reports
**MPS I**	Heparan sulfate, Dermatan sulfate	•Growth plate chondrocyte vacuolation •Failure of endochondral ossification [28] •Disorganized columnar structure [33]
**MPS** **II**	Heparan sulfate, Dermatan sulfate	•Failure of endochondral ossification [28] •FGF signaling deregulation during bone development [31] •Reduced calcification [28]
**MPS** **IIIA**	Heparan sulfate	•Reduced calcification [28] •Chondrocyte vacuolation [41]
**MPS** **IVA**	Keratan sulfate Chondroitin-6 sulfate	•Large, abnormal and vacuolated cartilage cells [28] •Failure of endochondral ossification [28] •Disorganized columnar structure [28] •Reduced calcification [28]
**MPS** **VI**	Dermatan sulfate Chondroitin-4 sulfate	•Growth plate chondrocyte vacuolation [34,35] •Failure of endochondral ossification [28] •Reduced calcification [28]
**MPS** **VII**	Heparan sulfate, Dermatan sulfate, Chondroitin-4 sulfate Chondroitin-6 sulfate	•Growth plate chondrocyte vacuolation [36,37,38,39] •Delay of development in primary and secondary centers of ossification [33] •Less ability to transit from the G1 to S phase and less ability to progress to mitosis or to exit the cell cycle [42] •Reduced length of limb bone and vertebrae [43]

**Table 2:** Biomarkers related to the bone system in MPS.

	Increased	Decreased
**Bone** **remodeling biomarkers**	Osteocalcin, BSAP, urinary PYD, and DYD (MPS I, II, VI) [60]	
**Pro-Inflammatory biomarkers**	IL1β, IL6, TNF-α,TGF-β, NO, and EGF (MPSII, IVA) [59] MMP-1,MIP-1 and MMP-9 (MPS IVA) [57]	MMP-2 (MPS IVA) [61]
**Transcriptional** **and genetic factors**	*SOX9* (MPS VII)*, COL2A1, NKX3-2* [42]	*RUNX2, ALPL, BGLAP, COL10A1,* osteopontin (*SPP1*) (MPSVII) [42]
**Expressed** **protein biomarkers**	MMP-2, -9,TIMP-1 (joint arthritis marker) [62] Lys-O-Gal/Lys-O-GalGlc (MPSI, II, IV) (collagen degradation marker) [63]	FOXA2, MMP13, PTCH1, PTH1R (MPS VII) [42] (chondrocyte maturation marker) TypeII A Collagen (MPSII, IVA) [64]
**Other new biomarkers** **in each MPS**	Collagen alpha-1 chain, Fatty acid-binding protein 5, Nidogen, cartilage oligomeric matrix protein, IGFBP7 (MPSI,IIneurological form) [61], βgalactosidase (MPSI, II neurological form, VI) [64] Glycosylated hydroxylysines (MPSIVA) A1T1, lipoprotein (a), SAA, clusterin and vitronectin (MPS IVA) [62] Protein HEG homolog1 (MPS VI) [64]	Fetuin-A (IVA) [63]
